# Vitamin A Deficiency in Children With Neurodevelopmental Disorders: Case Reports of Ocular and Urinary Tract Complications in Two Patients

**DOI:** 10.7759/cureus.85443

**Published:** 2025-06-05

**Authors:** Ryutaro Suzuki, Shinsuke Yoshizawa, Tomoka Kambe, Takashi Negishi, Kensuke Ohashi, Shintaro Nakao

**Affiliations:** 1 Department of Ophthalmology, Saitama Children's Medical Center, Saitama, JPN; 2 Department of Ophthalmology, Juntendo University School of Medicine, Tokyo, JPN; 3 Department of Urology, Saitama Children's Medical Center, Saitama, JPN

**Keywords:** avoidant and restrictive food intake disorder, keratomalacia, night blindness, urinary tract infections, vitamin a deficiency

## Abstract

Vitamin A deficiency (VAD) is a nutritional disorder that is predominantly observed in developing countries due to malnutrition. However, it can also occur in developed countries, particularly in children with neurodevelopmental disorders associated with avoidant and restrictive food intake disorder (ARFID) or malabsorption. The clinical manifestations of VAD include ocular complications, such as night blindness, xerophthalmia, and keratomalacia, as well as systemic effects, including impaired immunity, growth retardation, and increased susceptibility to infections. While there has been notable attention on the association between VAD and urinary tract infections, reports detailing their coexistence are limited. This report presents two pediatric cases of VAD in children with neurodevelopmental disorders who exhibited ocular and urinary tract symptoms. The first case involves a nine-year-old male patient who presented with progressive visual impairment, photophobia, and a medical history of urethral stenosis. A detailed examination revealed severe visual acuity loss, diffuse keratitis, optic nerve pallor, and keratin deposits in the bladder. The second case pertains to a six-year-old male patient who exhibited symptoms such as photophobia, recurrent urinary tract infections, and urethral keratosis. Upon examination, corneal leukoplakia and peripheral opacity were observed. A history of selective eating was observed in both patients, and serum vitamin A levels were used to confirm the diagnosis of VAD. Treatment with vitamin A and zinc resulted in significant improvement in ocular and urinary symptoms. Photophobia exhibited a marked improvement in both cases, although visual acuity recovery was limited in Case 1. These cases underscore the gravity of VAD, a condition that, though rare in developed countries, can manifest with severe and diverse symptoms, particularly in children with developmental disorders. The underlying mechanisms involve epithelial keratinization and immune dysfunction, which increase infection susceptibility. Early recognition and interdisciplinary collaboration are crucial for timely diagnosis and management. Clinicians are advised to consider VAD in patients presenting with ocular or systemic symptoms, particularly when urinary tract infections and dietary insufficiencies are concurrently present. These findings underscore the necessity of a multidisciplinary approach to address VAD, ensuring enhanced outcomes and the prevention of complications.

## Introduction

Vitamin A deficiency (VAD), a nutritional disorder prevalent in developing countries, has also been documented in developed countries. This is often due to extreme selective eating resulting from developmental or absorption disorders following gastrointestinal surgery [1-4]. This condition, characterized by insufficient intake or absorption of vitamin A, primarily manifests as eye complications, including night blindness, dry eye syndrome, and corneal softening, potentially leading to severe visual impairment and, in extreme cases, blindness. Systemically, it can lead to growth retardation, impaired bone and tooth development, hyperkeratosis of mucous membranes, dry skin, and an augmented susceptibility to infection due to a compromised immune system [5]. In recent years, its association with urinary tract infections has garnered attention.

In this report, we present a case series of two children diagnosed with developmental disorders who exhibited concomitant ocular and urinary tract infection symptoms attributable to VAD. While there has been a suggestion that vitamin D deficiency is associated with urinary tract infections, there have been only a few reports of the coexistence of VAD and urinary tract infections [6-8]. The objective of this report is twofold: first, to raise awareness of VAD and its complications, and second, to emphasize the importance of interdisciplinary cooperation in the treatment of VAD.

## Case presentation

Case 1

A nine-year-old boy patient presented to the hospital complaining of progressive visual impairment and photophobia since the age of 5. His past medical history included neurodevelopmental disorders and urethral stenosis. The initial onset of visual impairment was observed at the age of five years and nine months, with a subsequent rapid progression. The patient exhibited behaviors such as excessive eye rubbing, which became particularly prominent after the age of 7.

**Figure 1:**
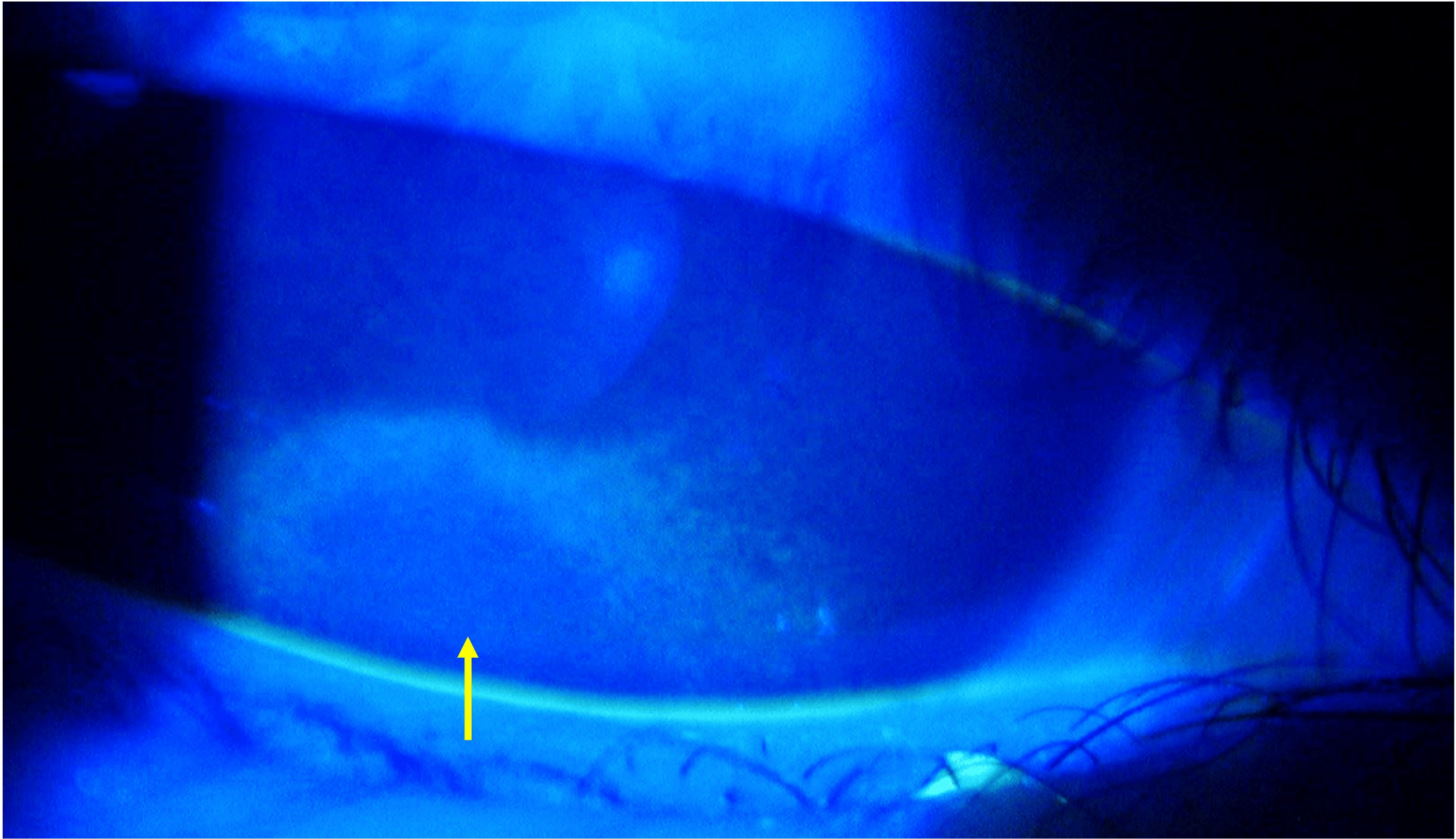
Fluorescein-stained image of the right eye at initial examination. Arrow indicates findings of diffuse keratitis.

**Figure 2:**
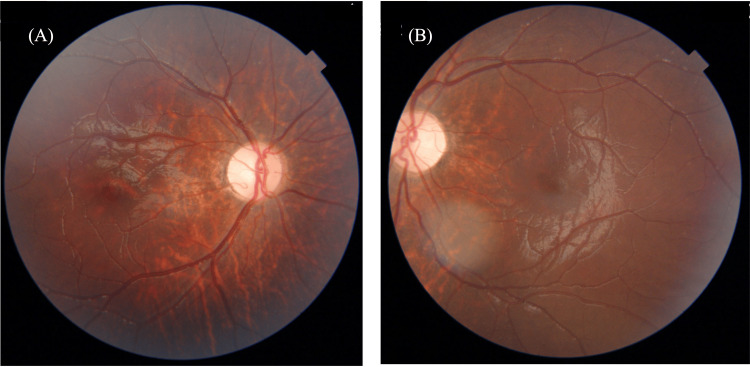
Fundus photograph at initial examination. (A) Image of the right eye and (B) image of the left eye. Pallor of the optic papillae was observed in both eyes.

**Figure 3:**
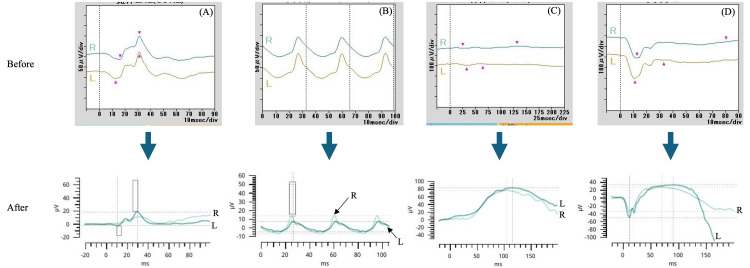
Comparison of full-field electroretinograms before and after both vitamin A and zinc supplementation. (A-D) Images of cone ERGs, flicker ERGs (ISCEV), rod ERGs, and flash ERGs, respectively. Prior to treatment, the rod response was lost, and the maximal response showed a negative type. After treatment, they improved. ERG: electroretinogram; ISCEV: International Society for Clinical Electrophysiology of Vision.

**Figure 4:**
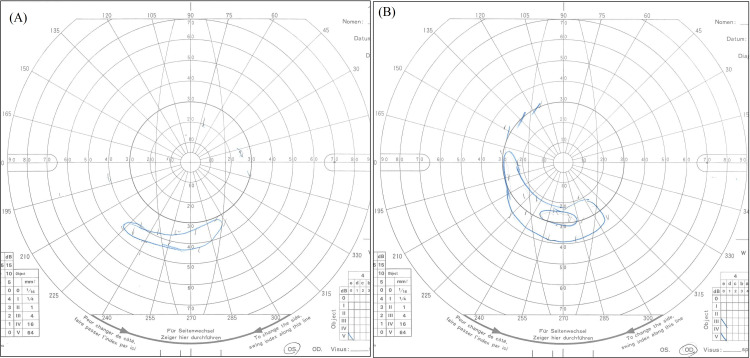
Results of Goldmann visual field test at initial examination. (A) The visual field of the right eye and (B) the left eye. Arrows indicate that only the peripheral field of view remains.

At the time of the initial examination, the patient exhibited a marked deterioration in visual acuity, 0.01 (n.c.) in both eyes. Further examination revealed significant corneal changes, including Bitot’s spots and diffuse keratitis, as clearly shown by fluorescein staining (Figure 1). A fundus examination revealed bilateral optic nerve pallor especially on the temporal side (Figure 2). Full-field electroretinography showed the disappearance of the rod response and a negative maximal response pattern (Figure 3). The Goldmann visual field test revealed that only the peripheral visual field was preserved (Figure 4).

In this case, the patient was treated for urethral stenosis at the age of 8, but the difficulty in urinating recurred. A cystoscopy revealed the presence of keratin deposits in the bladder, the etiology of which could not be ascertained. Consequently, a comprehensive evaluation was conducted, encompassing a review of pertinent medical history, a thorough physical examination, and an analytical assessment of blood vitamin A levels. This multifaceted approach led to the substantiated diagnosis of VAD. He was diagnosed with VAD keratoconjunctivitis and treated with oral vitamin A (10,000 units) and zinc (34 mg) daily for 30 days.

**Figure 5:**
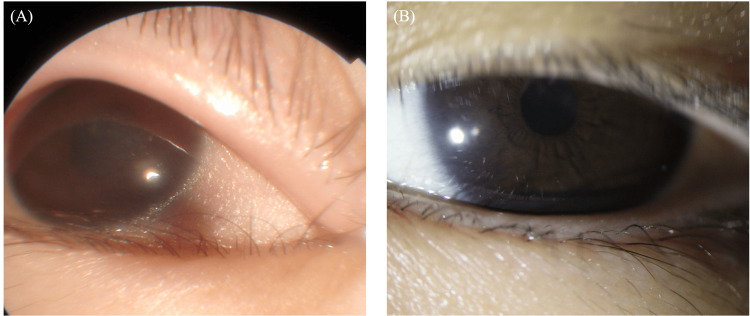
Conjunctival images before (A) and after treatment (B) approximately two years after the initial visit. Before treatment, keratinization of the conjunctiva was observed, but after treatment, improvement was observed.

In this case, improvement of corneal findings began one month after vitamin A administration, and at four months, the Bitot's spots disappeared, and the cornea became clear (Figure 5). The final visual acuity was confirmed in both eyes when assessed with hand motions. Although the numerical value of the test result worsened, the patient was able to write in her notebook at school, suggesting that she could actually see better than when her visual acuity was confirmed by hand movement (Figure 3).

Case 2

**Figure 6:**
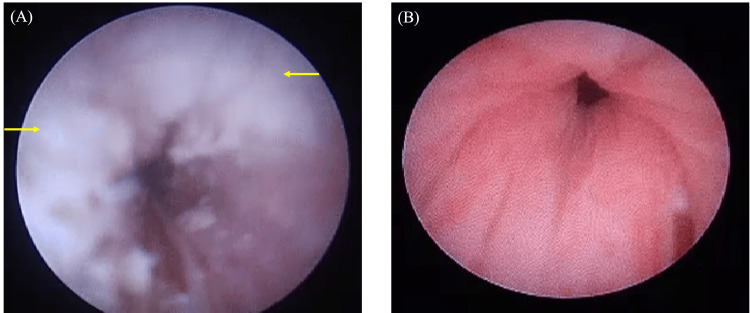
Cystoscopic images at initial presentation and after vitamin A and zinc replacement therapy. (A) Image at the initial examination and (B) image after treatment. In (A), the arrows indicate keratin deposits in the urethra, which have disappeared after treatment.

A six-year-old boy patient presented at the hospital with a primary complaint of photophobia. His past medical history included a diagnosis of autism spectrum disorder (ASD) and recurrent urinary tract infections. He had a medical history marked by urethral keratosis and recurrent keratitis since the age of 2. A thorough examination of the anterior eye segment of the right eye revealed the presence of corneal leukoplakia and peripheral opacity. Given the patient's history of selective eating and the presence of cystoscopy findings similar to those observed in Case 1, in consultation with the urology department, we measured the patient's serum vitamin A levels and found them to be below detectable levels (Figure 6).

**Figure 7:**
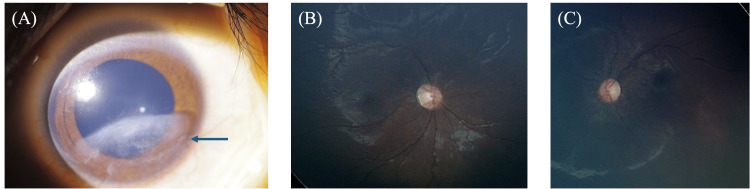
Anterior and fundus photographs after treatment. (A) Photograph of the anterior segment (state the eye) after treatment. The arrows indicate that the opacity in the central part of the cornea remains, but the opacity in the ring area has improved. (B, C) Fundus photographs after treatment. Atrophy of the optic papilla remains in both eyes.

Final visual acuity was 1.6 cy/cm in the right eye on Teller Acuity Cards. The examination of the left eye could not be performed due to refusal. In this case, photophobia improved, and the patient was able to walk one month after initiating oral administration of 10,000 units of vitamin A per day. Six months later, the superficial keratitis was negligible. However, corneal opacity remained in the right eye and optic disc atrophy in both eyes (Figure 7).

## Discussion

The prevalence of VAD is particularly pronounced in developing countries, where it is primarily driven by malnutrition. However, in this particular instance, the condition has also been observed in developed countries, affecting children with neurodevelopmental disorders due to malnutrition resulting from ARFID [9]. Specifically, in children with neurodevelopmental disorders, urinary tract infections have been reported, and the pathophysiological mechanism has been identified as the keratinization of epithelial tissue and the decline in immune function due to VAD [8]. Vitamin A plays an important role in maintaining epithelial tissue and regulating the immune system. It has become evident that its deficiency causes a decline in mucosal barrier function and abnormal immune responses [8].

It has been reported that around 5% of patients with eating disorders have a comorbid ASD and that around 60% of parents with children with pervasive developmental disorders experienced strong preferences in food [10,11]. In particular, children with autism tend to favor carbohydrates, consume less fat and protein, and are more likely to be deficient in calcium, phosphorus, iron, vitamin A, and B vitamins than typically developing children [12].

According to a previous study, VAD-induced ocular dryness causes conjunctival keratinization due to decreased conjunctival goblet cell and mucin production, and it has been reported that partial goblet cell regeneration and a marked decrease in conjunctival keratinization were observed one week after the start of VitA administration and that goblet cells regenerated and both the cornea and conjunctiva normalized after two weeks [13]. In this case, improvement of the corneal findings was also observed early after treatment, but opacity remained, and complete visual recovery was not achieved.

In experimental animal models, it has been confirmed that vitamin A-deficient mice exhibit an increase in the frequency of urothelial squamous metaplasia and ascending urinary tract infections. The elucidation of the pathophysiology of ocular complications and urinary tract infections in this disease is an important clinical issue [5]. Recent studies have indicated that vitamin A supplementation in patients with VAD may enhance the therapeutic effect of antimicrobial therapy for urinary tract infections [14]. Moreover, experimental animal models have demonstrated that VAD during pregnancy contributes to abnormal development of the urinary system in the fetus. It has been posited that this deficiency may act as a risk factor for urinary tract infections throughout life [5]. Consequently, it is becoming evident that VAD possesses significant clinical implications, extending beyond its conventional role as a nutritional disorder. It is now recognized as a factor that can compromise immune function, thereby increasing susceptibility to infection.

The symptoms associated with VAD are sometimes considered to have an unknown etiology or to be intractable by departments other than ophthalmology [15]. In the two cases reported here, the urologist had confirmed the presence of bladder findings of unknown etiology but had not suspected a link with VAD. However, by suspecting VAD based on the typical eye symptoms and initiating treatment, a favorable outcome was achieved, marked by the improvement of the urinary tract infection. The diagnosis of VAD was made expeditiously through collaborative efforts and information exchange with the relevant departments.

## Conclusions

In children diagnosed with developmental disorders, the anterior segment of the eye is often challenging to examine and assess for visual function in the presence of photophobia. When a patient presents with symptoms suggestive of VAD, a review of pertinent medical history, including the presence of urinary tract infections and dietary habits, can facilitate a rapid diagnosis. Complications arising from VAD manifest in a variety of symptoms, including, but not limited to, urinary tract infections. Consequently, effective communication and collaboration with relevant departments are paramount to ensure comprehensive management of these patients.
